# Motivation Analysis of Online Green Users: Evidence From Chinese “Ant Forest”

**DOI:** 10.3389/fpsyg.2020.01335

**Published:** 2020-06-30

**Authors:** Bo Chen, Yi Feng, Jinlu Sun, Jingwen Yan

**Affiliations:** ^1^Institute for Finance and Economics, Central University of Finance and Economics, Beijing, China; ^2^Mental Health Center, Central University of Finance and Economics, Beijing, China; ^3^School of Psychology, Beijing Normal University, Beijing, China; ^4^School of Humanities and Social Sciences, Beihang University, Beijing, China; ^5^China Academy of Public Finance and Public Policy, Central University of Finance and Economics, Beijing, China

**Keywords:** climate change, digital technology, international cooperation, motivation, Ant Forest

## Abstract

Environmental protection activities based on digital technology have cultivated many online green users (OGUs) and may become a critical means to combat global climate change. This paper explores individuals’ motivation to participate in online environmental protection activities and whether the activities have significantly increased individuals’ intention to participate in global collaboration on climate change. Taking Ant Forest as an example, this paper first summarized 14 trigger reasons for users’ participation in online environmental protection activities through interviews, then surveyed 600 OGUs through questionnaires, and studied the behavioral motivation from the four dimensions of environmental awareness, social motivation, online immersion, and global cooperation intention by using a structural equation model. The study found that both environmental awareness and social motivation had significant positive promotional effects on OGUs’ online immersion, and environmental awareness was higher than social motivation. Environmental awareness as a long-term motivation is conducive to the achievement of long-term climate goals, and social motivation is focused on short-term entertainment functions. There is a significant positive interactive relationship between environmental awareness and social motivation under the effect of digital technology, which jointly promote the improvement of OGUs’ online immersion, and online immersion is conducive to enhancing OGUs’ global cooperation intention. This study demonstrated that digital technology can effectively improve individuals’ intention to protect the environment and found a means to quickly identify the best OGUs (most willing to participate in global cooperation), which provided a new opportunity to inspire greater public participation in the global action against climate change.

## Introduction

Human activities have resulted in an increase in greenhouse gas concentrations that has led to climate change and its uncertain consequences (Meehl et al., 2000; Gifford, 2011; Bai et al., 2018; Holmgren et al., 2019). Although this scientific consensus has been reached, reducing emissions under this substantial environmental pressure requires a gradual process (Figueres et al., 2017); thus, the public and industry need to be aware of the seriousness of the problem and participate in mitigation (Oreskes, 2004).

Experiments have demonstrated many factors that affect the public engagement of climate change, such as cognitive bias (Lazarus, 2008; Holmgren et al., 2018), lack of knowledge (Sterman and Sweeney, 2007), negative footprint illusion (Holmgren et al., 2019), and even the experience of extreme weather (Bergquist et al., 2019; Marshall et al., 2019), leading to public disregard for this problem (Mazar and Zhong, 2010; Sorqvist and Langeborg, 2019), which is the so-called “psychological distance of climate change” (McDonald et al., 2015). Recent literature shows a negative relation between psychological distance of climate change and pro-environmental behavioral intentions (Sacchi et al., 2016). Hence, scholars have called for effective means of communication to reduce the psychological distance of climate change (Brugger et al., 2016; Jones et al., 2017; Ejelov et al., 2018).

Fortunately, digital technology might be an effective strategy to increase cooperation to address climate change by reducing the psychological distance. On one side, digital technologies represented by the Internet are changing the behavioral patterns of individuals’ participation in environmental protection movements, and possibly becoming an effective policy tool (Mckenna and Bargh, 2000; Engelberg and Sjoberg, 2004; Bierhoff and Vornefeld, 2004). On the other side, the rapid advancement of digital technology has enabled the public to understand climate change in a vivid manner by offering convenient instruments, such as mobile apps, and participate in global environmental protection cooperation, which is vital for global warming mitigation (Bai et al., 2018; Mentes, 2019). Therefore, it is expected that digital technology can promote online activities to help individuals cope with climate change and increase their willingness for global cooperation by reducing psychological distance. However, research regarding how digital technologies improve individual’s engagement in climate change activities is relatively sparse and most extant studies are in experimental environment and there is little analysis of the real case.

The purpose of this paper was to explore whether the application of digital technology has improved the public’s awareness of the environment and willingness to participate in global responses to climate change, and how this mechanism works with the case of the online green users (OGUs) on Ant Forest app, the world’s largest online public environmental platform. Since the launch of Ant Forest app by Alipay in 2016, it has cultivated nearly 500 million OGUs and is—to date—the only environmentally aligned fintech app to achieve mass scale. This success is due to the access it provides to new sets of data and digital tools that incentivize green behaviors. OGUs use the Alipay platform to pay for everything, for example, groceries, food deliveries, bus and train tickets, gasoline, and water and electric bills. Additionally, enabled by artificial intelligence, Alipay provides users with real-time feedback on concrete online actions and behaviors they can take to reduce their individual carbon footprint. Any carbon-light behavioral changes are immediately rewarded with green-energy points on the Ant Forest platform. If the user chooses to “make a game of it,” he or she can use the Ant Forest app to monitor the accumulated energy points to aggregate them into a “virtual tree” on the app. A gamification feature of Ant Forest enhances user engagement by having them continually accumulate the additional energy points necessary to sow, water, and care for their virtual tree until it reaches maturity on the screen, and users can give friends energy or collect friends’ energy. Once the virtual tree has grown, Alipay matches the virtual tree with the planting of a real tree or the protection of a conservation area as a reward for the continuous reduction in the user’s individual carbon footprint. The trees are planted in partnership with established non-governmental organizations (NGOs) and funded directly by Alipay’s parent company, Ant Financial Services Group. Users can earn green certificates and view images of their trees in real time through satellites.

Based on this context, this study aims to explore the behavioral motivation of OGUs and then discuss whether online green activities encourage their enthusiasm for environmental issues as well as whether their online immersion originates from increased environmental awareness. Moreover, it is valuable to explore OGUs’ intention to participate in global climate change cooperation. The marginal contribution of this study is to reveal how large-scale digital technology affects people’s green behavior patterns and increase the willingness to cooperate globally, which enriches the subject of environmental psychological distance.

## Materials and Methods

### Participants

This study consists of a cross-sectional design and was conducted from 1 to 30 October 2019. Random sampling was used to collect the OGU samples through the Internet in China. Participants in the survey had to meet the following criteria: (1) be Chinese Internet users; (2) users on Ant Forest; and (3) able to understand Chinese.

### Survey Design and Measures

For this study, we designed a set of OGU-oriented questionnaire surveys. First, prior to this survey, we conducted semi-structural interviews, and 51 OGUs were individually interviewed regarding their usage motivation, frequency of use, public welfare behavior, data privacy, and comments. As a result, 14 triggers were extracted, which reflected the people’s complex behavioral motivation. Then, a set of online questions was designed based on these 14 triggers. The participants clicked on a button to verify their consent to complete an online survey named the “Ant Forest User Survey.” The survey comprised a series of questions. Demographic measures included age, gender, region, income, and environmental satisfaction. The 14 triggers were as follows:

Trigger 1: Health situation. Using an app that rewards a carbon-light lifestyle can result in positive health outcomes for the user (e.g., if driving is replaced by walking).
Trigger 2: Environmental situation. If a user lives in an area marked by environmental degradation and/or pollution shifting to a greener lifestyle can be triggered.
Trigger 3: Entertainment and socializing. The fun style and chance to interact with others can be a trigger to engage on the app.
Trigger 4: Curiosity and education. Interested in acquiring the learnings from using the app.
Trigger 5: Globalization. Motivated by an understanding that climate change and deforestation have global effects that can impact directly on the user’s life.
Trigger 6: Fun interaction of carbon footprint and tree planting. Motivated by a compelling fun function related to carbon footprint and/or tree planting.
Trigger 7: Interactive social elements. Motivated by the social networking, cooperation, competition, and/or other community and competition elements.
Trigger 8: Sense of honor and reputational enhancement. Motivated by a desire to see oneself and to be seen by others as making a positive contribution to society.
Trigger 9: Zero transaction cost. Using the app because there are no costs to the user.
Trigger 10: Incentive level. Engage because one can potentially gain access to advantages.
Trigger 11: Credibility of carbon footprint calculations and of tree planting programs.
Trigger 12: Data privacy protection. Motivation or lack thereof because behavioral data analysis and translation into personal carbon footprints require algorithms based on access private data.
Trigger 13: Interest in nature and biodiversity. Motivated by the ability to contribute to positive impacts on nature.
Trigger 14: Social responsibility. User’s own level of social responsibility awareness.

The above 14 triggers reflect the complex driving forces of OGU users, and digital technology integrates these triggers to form the OGU’s behavior pattern. In the Ant Forest model, the behavior patterns of OGUs can be described through four dimensions: social motivation (Mckenna and Bargh, 2000; Bargh and Mckenna, 2004; Engelberg and Sjoberg, 2004; Simpson and Willer, 2015), environmental awareness (Fehr and Gintis, 2007; Boyd and Richerson, 2009), online immersion (Bierhoff and Vornefeld, 2004), and global cooperation intention (Lazarus, 2008; Ejelov et al., 2018). In addition, demographic characteristics are considered (Van Vugt et al., 2007; Charness and Rustichini, 2011; Masod and Chin, 2014; Branasgarza et al., 2018; Parrish et al., 2019; Cigarini et al., 2020).

#### Social Motivation

**FIGURE 1:**
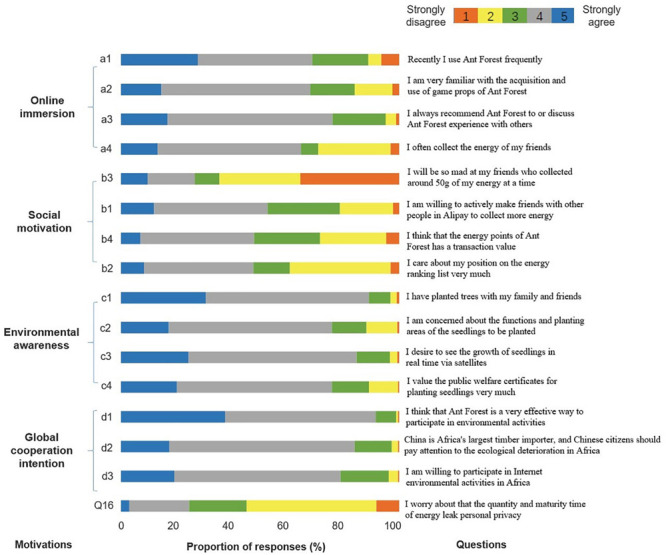
Each motivation was assessed with three to four items including online immersion (a1–a4), social motivation (b1–b4), environmental awareness (c1–c4), and global cooperation intention (d1–d3). Q16 was eliminated by exploratory analysis. For each item, participants indicated their level of agreement from 1 (*strongly disagree*) to 5 (*strongly agree*).

Social motivation refers to importance of the social networking and fun interaction elements (i.e., energy points, ranking, interaction with others to collect more energy, and concern over others collecting/stealing their energy). Social motivation is a very comprehensive factor, and multiple triggers may be related to it. Therefore, four items with a five-point scale, from “strongly disagree” to “strongly agree,” were used to assess social motivation. The four items (see Figure 1) were related to social skills (e.g., “I am willing to actively make friends with other people in Alipay to collect more energy”), recognition by the network (e.g., “I care about my position on the energy ranking list very much”), incentive level (e.g., “I will be mad at my friends who have collected around 50 g of my energy at a time”), and benefits (e.g., “I think that the energy points of Ant Forest have a transactional value”). Responses to the four items created a composite score for social motivation (Cronbach’s α = 0.64; AVE = 0.32; CR = 0.93), with higher scores indicating higher motivation levels.

#### Environmental Awareness

Environmental awareness refers to importance of real and verified impact (improving the environment as a public good through real tree planting), access to visualizing this impact, and receiving certificate for planting real seedlings. Similar to the approach to social motivation, four items with a five-point scale, from “strongly disagree” to “strongly agree,” were used to measure environmental awareness. The items included two triggers: recognition by the network and credibility, such as “I have planted trees with my family and friends” and “I am concerned about the functions and planting areas of the seedlings to be planted.” Responses to the four items created a composite score for environmental awareness (Cronbach’s α = 0.69; AVE = 0.39; CR = 0.95), with higher scores indicating higher levels of environmental awareness.

#### Online Immersion

Online immersion refers to frequency of use and length of time spent on the Alipay Ant Forest platform. Online immersion was assessed by four items with a five-point scale, from “strongly disagree” to “strongly agree.” Online immersion contains four triggers: entertainment, game ability, curiosity, and incentive level, such as “I use Ant Forest frequently” and “I am very familiar with the acquisition and use of game props in Ant Forest.” Responses to the four items created a composite score for online immersion (Cronbach’s α = 0.73; AVE = 0.38; CR = 0.94), with higher scores indicating more online immersion.

#### Global Cooperation Intention

Global cooperation intention refers to willingness to engage in tree planting or preservation of biodiversity activities outside of China. This dimension is measured by users’ willingness to participate in forest conservation in Africa. The variable “global cooperation intention” was constructed as an output variable through three triggers: social responsibility, concern for nature, and globalization. The items consisted of three items (i.e., “I think that Ant Forest is a very effective way to participate in environmental activities”; “China is Africa’s largest timber importer, and Chinese citizens should pay attention to the ecological deterioration in Africa”; and “I am willing to participate in Internet-based environmental activities in Africa”). The composite score of “global cooperation intention” was created (Cronbach’s α = 0.70; AVE = 0.47; CR = 0.97), with higher scores indicating a stronger global cooperation intention.

### Data Analysis

First, we investigated the dimensionality of user behavior through an exploratory factor analysis (EFA). We extracted factors using principal component analysis and rotated the solution by means of varimax orthogonal rotation, leading to correlated factors. Factor solutions were interpreted with salient factor pattern loadings of 0.45 and higher. Moreover, utilizing items assigned uniquely to factors, we calculated Cronbach’s alpha for each factor. Second, according to the result of the EFA, we employed the Shapiro–Wilk test to examine the normality of continuous variables. Spearman correlations between the variables measured above were calculated if the continuous variables were not normally distributed; otherwise, Pearson correlations were calculated. Third, we constructed four variables as latent variables. The hypothesized mediating effect of online immersion was examined by structural equation modeling (SEM) using Amos (version 24) software. The goodness of fit was assessed by computing the comparative fit index (CFI), goodness-of-fit index (GFI), adjusted GFI (AGFI), root-mean-square error of approximation (RMSEA), and the standardized root mean residual (SRMR) (Karl and Dag, 1982; Hu and Bentler, 1998). The acceptable levels of good-fit model parameters are CFI > 0.90, GFI > 0.90, AGFI > 0.90, RMSEA < 0.08, and SRMR < 0.08. The significance level was set 0.05 in this study.

## Results

### Demographic Characteristics

A total of 1005 questionnaires were randomly issued, with 30 invalid samples removed; the response rate was 97.01%. Because not all Internet users are OGUs, filter conditions were set to screen out OGUs using the item “Please indicate whether you are using Ant Forest or not.” The final 600 OGU participants were filtered from the remaining 975 samples. The proportion of women was 63.5% of the total, and 90.1% of those surveyed were aged between 18 and 40 years old. Most of the participants in the sample were from Eastern China (59.2%), and most had an annual income below CNY 200,000 (92.0%). In terms of local environmental satisfaction, the “general” and “satisfied” populations accounted for 41.7 and 43.8%, respectively, indicating that most users have a high level of satisfaction regarding the environment.

### Exploratory Factor Analysis

An EFA was conducted, where the KMO value (0.88) was greater than 0.8, and the Bartlett test of sphericity was passed, indicating that the construct validity of the questionnaire was acceptable. The responses related to privacy (“I worry that the quantity and maturity time of energy threaten my personal privacy”; Q16) revealed that OGUs were not very concerned about privacy disclosure and thus had to be deleted, indicating that user privacy is not a significant affecting factor for Ant Forest user behavior. Principal component analysis was conducted for the remaining 15 questions and indicated that the cumulative variance contribution rate (58.08%) of four factors was higher than 55%; thus, four factors could be used to construct the model. The rotated factor matrix differentiated the latent variables of four dimensions: online immersion (a1–a4), social motivation (b1–b4), environmental awareness (c1–c4), and global cooperation intention (d1–d3) (see Table 1).

**TABLE 1:** Factor loadings for the items in the questionnaire.

	Factor			
Items	1	2	3	4
a1. Entertaining	0.84			
a2. Game ability	0.73			
a4. Incentive level of collect energy	0.58			
a3. Curiosity	0.47			
b3. Incentive level by others		0.77		
b2. Recognition of position ranking		0.68		
b4. Transaction value		0.58		
b1. Social skills		0.50		
c4. Certificates for planting			0.73	
c3. Credibility of growth			0.68	
c2. Credibility of function			0.61	
c1. Recognition of close others			0.52	
d2. Concern for nature				0.82
d3. Global activities				0.78
d1. Social responsibility				0.52

*Factor 1: Online immersion; Factor 2: Social motivation; Factor 3: Environmental awareness; Factor 4: Global cooperation intention.*

### Correlations Analysis

We found that all continuous variables were non-normally distributed, so Spearman correlation analyses were conducted. Significant positive correlations were found between social motivation, environmental awareness, online immersion, and global cooperation intention (see Table 2). No significant correlations were found between the demographic variables and the interested variables.

**TABLE 2:** Spearman’s correlations between main variables.

	1	2	3	4	5	6
1 Gender	1					
2 Age	−0.15**	1				
3 Social motivation	0.03	0.00	1			
4 Environmental awareness	–0.01	–0.04	0.35**	1		
5 Online immersion	–0.01	0.05	0.30**	0.36**	1	
6 Global cooperation intention	0.04	–0.05	0.31**	0.47**	0.32**	1

**p < 0.01. The gender was coded as “1 = male,” “2 = female.”

### Structural Equation Modeling

**FIGURE 2:**
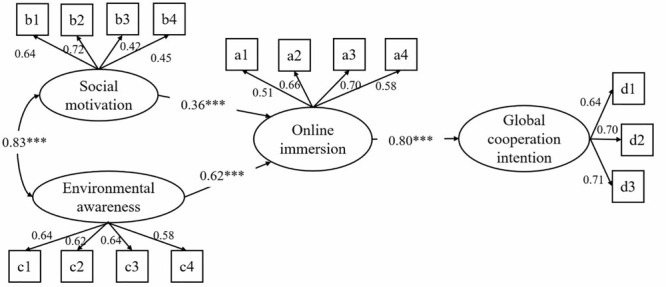
Parameter estimation of the structural equation model. All parameter estimates are all significant at the significance level of 0.001. The results showed that social motivation and environmental awareness jointly influenced online immersion and further determined the global cooperation intention.

We used SEM to examine whether or not social motivation and environmental awareness could significantly affect online immersion and whether online immersion could further promote global cooperation intention. Figure 2 demonstrates the final SEM model, which fits well with the data (χ^2^/*df* = 3.638, CFI = 0.963, GFI = 0.931, AGFI = 0.903, RMSEA = 0.066, SRMR = 0.074). This model showed that environmental awareness and social motivation jointly stimulate users’ online immersion. Users’ social motivation has a positive effect on online immersion (β = 0.36, *p* < 0.01). Furthermore, environmental awareness has a positive effect on Ant Forest activity immersion (β = 0.62, *p* < 0.001), which is higher than social motivation and indicates that environmental awareness has a greater effect than social motivation. Users with higher levels of online immersion have higher levels of global cooperation intention (β = 0.80, *p* < 0.001), and this positive promotional effect indicates that active users of Ant Forest are more convinced of the environmental benefits of online activities. For example, these users believe that the remediation of the ecological deterioration in Africa is necessary, and they are more willing to participate in online non-profit tree planting activities in Africa. This result reveals the inherent driving factors of OGUs’ behavior patterns.

### Quick Means to Identify Target Users Through Screening

**FIGURE 3:**
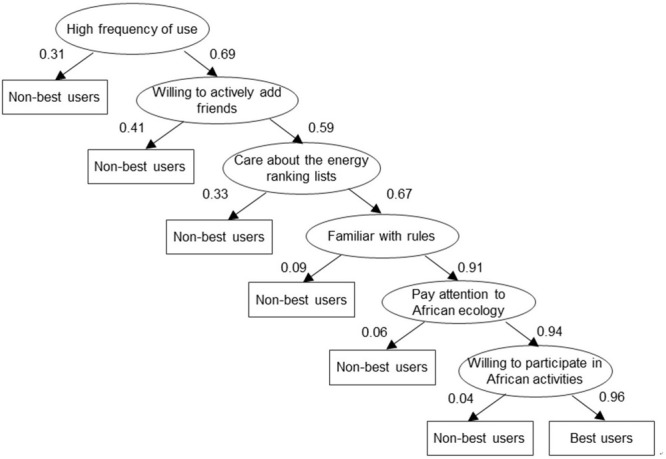
Six steps to quickly identify target users with high global cooperation intention. These six steps represent the six questions in the questionnaire (a1, b1, b2, a2, d2, and d3).

**FIGURE 4:**
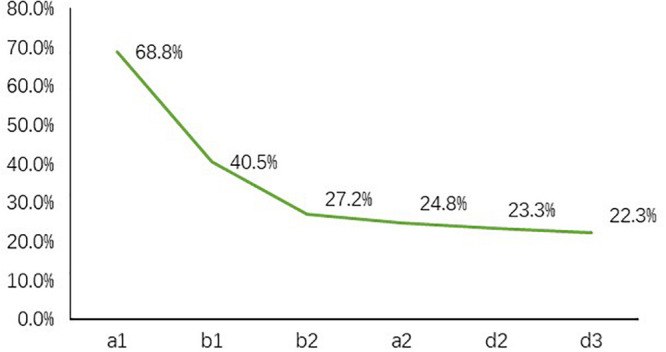
The shape of the OGU best user searching curve indicates that it becomes smoother after the third step.

When formulating a digital climate policy, policymakers need to find the most likely supporters (“best users”) from the huge Internet users. One of the most significant concrete outcomes from the research is the validation of a rapid screening tool that accurately identifies potential best users (i.e., those most likely to be persuadable to act on international cooperation) through six indicators that had been refined through the course of the research. The resulting “decision tree” tool accurately identifies potential best users across different segments (see Figure 3). Application of the tool demonstrates that more than one fifth (22.3%) of the Chinese users sampled are willing to participate in planting trees to solve the problem of deforestation in Africa. Crucially, the convergence starts to slow after the third step. Thus, as long as the first three steps are completed, the relatively ideal screening (27.2% versus the finer winnowing to the aforementioned 22.3%) can still be achieved but at significantly reduced cost of user screening (see Figure 4). When the number of users is extremely large, reducing one screening step means saving huge search costs.

## Discussion

The study confirms the notable potential of digital technologies to encourage public participation in mitigating global climate change. Extant studies have suggested that individuals’ understanding of climate change is limited; that is, individuals do not recognize the seriousness of the problem, have no strong sense of improving the situation, and may overpraise the behavior of slight emission reduction (Sterman and Sweeney, 2007; Lazarus, 2008; Mazar and Zhong, 2010; Holmgren et al., 2019; Sorqvist and Langeborg, 2019). These misconceptions may result from the large psychological distance between humans and climate change, resulting in a lower intention to participate in environmental protection activities (Brugger et al., 2016; Jones et al., 2017; Ejelov et al., 2018). However, this study demonstrates that under the context of digital technology, individuals can see the real-time planting of seedlings through satellites, care for seedlings in simulated situations online, and earn digital green certificates; thus, public participation in climate change mitigation activities can be significantly increased after these aspirations are fulfilled.

This study also demonstrates that environmental awareness and social motivation had a significant positive promotional effect on OGUs’ online immersion, which has not been illustrated by current studies. Environmental awareness was higher than social motivation, indicating that environmental awareness as a long-term motivation is more conducive to the achievement of long-term goals (Ejelov et al., 2018; Marshall et al., 2019) and that social motivation is more focused on short-term entertainment functions and reflects more satisfaction with completion and interaction than that of social and game attributes, which effectively motivate individuals to take concerted action against climate change (Nyborg et al., 2016; Xie et al., 2018). Notably, this study observes a significant positive interactive relationship between environmental awareness and social motivation, and both improve OGUs’ online immersion. This finding indicates that users’ long-term and short-term motivations are not isolated, but this phenomenon may occur because digital technology makes it easier for individuals to build trust and collaboration (Bierhoff and Vornefeld, 2004; Engelberg and Sjoberg, 2004).

Studies have demonstrated that achieving global environmental cooperation is difficult because of the large differences among regions, the different environmental problems they face, and the possible conflicts of interest (Lazarus, 2008). However, this study demonstrates that online immersion had a significant promotional effect on global cooperation intention, indicating that online activities have a real effect on individuals’ intention to participate in global climate cooperation. Therefore, extensive global cooperation can be achieved by visualizing remote environmental problems through digital technology, strengthening the link between climate deterioration and the participants, and encouraging more individuals to participate through more vivid and notable activities.

The study has the following limitations: First, there may be sample bias in collecting questionnaires from the Internet, which can be further improved by collecting offline questionnaires. Second, this study is aimed at Chinese users and does not consider the moderating effects of culture variables, so cross-cultural research can be conducted on OGUs in other countries. Third, this study uses a self-edited questionnaire, which may have certain limitations, for which the interview can be used to further validate the questionnaire. Fourth, the questionnaire method can only explore correlations, and experimental methods can be used to study causality in the future.

## Applied Application

The study in this paper has demonstrated that a combination of environmental awareness and social motivation through digital technology can encourage individuals to participate in global climate change mitigation activities in an intuitive, convenient manner. This environmental tool has not been fully exploited. Because climate change is caused by human activities, its solutions are closely linked to human daily activities; thus, further research on the psychology and behavior of OGUs would advance the field. Digital technology can crowd-in engagement from the ground up in manners that multilateral top-down approaches have not managed. Individuals’ online environmental protection activities’ footprint, combined with digital technology, can be effectively collected, and their intrinsic motivation can be explored based on big data, which further promotes the efficient development of environmental protection activities and more effectively motivates OGUs to participate in mitigating climate change. However, careful design is required to ensure that the solutions trigger behaviors that make individuals feel committed to resolving the problem and thereby willing to invest time in these solutions through engagement on a digital platform.

## Data Availability Statement

The datasets used during this study are available from the corresponding author YF (fengyi@cufe.edu.cn) on reasonable request.

## Ethics Statement

The studies involving human participants were reviewed and approved by the Central University of Finance and Economics. The participants were informed regarding the purpose and procedures of this survey via instructions at the head of the questionnaire. Informed written consent was provided on the first page of the questionnaire for all the participants.

## Author Contributions

BC was responsible for the overall research ideas, model design, and thesis writing. JS was responsible for literature review writing and data collection and processing. JY was responsible for model optimization and discussion of research conclusions. YF was responsible for manuscript revision and tables re-making.

## Conflict of Interest

The authors declare that the research was conducted in the absence of any commercial or financial relationships that could be construed as a potential conflict of interest.
